# Supplementary material for: Are carbon emissions trading and green financial instruments synergistic? -Comprehensive quantitative research based on content analysis
Source: PLoS One. 2024 Mar 7;19(3):e0298601. doi: 10.1371/journal.pone.0298601 (PMC10919601; doi:10.1371/journal.pone.0298601)
Supplement: S1 Data — (ZIP) [file pone.0298601.s001.zip › Data/地级市Z_F工作报告.zip!/─┌├╔╣┼/░n═╖╩╨/└┤╫╘╣1⁄2╓┌║┼í╛┬φ┐╦╩2╛▌═°í┐_2009.docx]

各位代表：

现在，我代表市人民政府向大会作工作报告，请予审议，并请各位政协委员和其他列席人员提出意见。

一、2008年工作回顾

2008年是本届政府的开局之年。在中共包头市委的领导下，市政府以邓小平理论和“三个代表”重要思想为指导，全面落实科学发展观，积极应对各种困难和挑战，圆满完成了市十三届人大一次会议确定的任务。预计全年地区生产总值完成1700亿元以上，增长20%左右。财政总收入完成205.2亿元，增长25.9%。预计城乡50万元以上固定资产投资完成1100亿元左右，增长35%左右；社会消费品零售总额达到515亿元，增长23.5%；城镇居民人均可支配收入达到21000元左右，增长17%左右；农牧民人均纯收入达到7200元左右，增长17%左右；城镇登记失业率3.87%，人口自然增长率4.67‰。预计单位生产总值能耗降低6.5%，二氧化硫和化学需氧量排放量分别控制在18.46万吨和2.74万吨以内，将全面完成自治区下达的节能减排任务。向全市人民承诺的20件40项好事实事全部落实，有效解决了一批人民群众关心的热点难点问题。

2008年也是改革开放30周年。30年改革开放的巨大成就，进一步鼓舞和激励了全市人民建设包头的热情，增强了推动科学发展、富民强市的合力，建设富裕民主文明和谐新包头迈出新的步伐。2008年，我市以地级城市最高分蝉联全国文明城市荣誉称号，奥运火炬包头段传递活动圆满完成。实现“全国双拥模范城”六连冠和“全国科技进步先进城市”五连冠，被评为“2008年中国制造业名城”、“中国城市信息化50强”、“2008年度最具影响力的品牌城市”和“中国优秀创新型城市”。

一年来，我们主要抓了以下工作：

（一）全面实施工业强市战略，提升工业经济综合实力。预计规模以上工业增加值完成770亿元左右，增长26%左右。优势特色产业支撑带动能力增强。以培育壮大钢铁、铝业、装备制造、电力、稀土等支柱产业为重点，新建和续建了214个5000万元以上工业重点项目。稀土永磁驱动管道输运系统集成开发、汇全兆瓦级风力发电机组、中复合风电叶片设备制造、中国重汽宏昌天马专用车生产线、华远10万吨不锈钢冷轧板、鑫普4000吨贮氢合金粉等项目加快实施；包钢宽厚板生产线及2×15万千瓦燃气轮联合发电机组、包铝综合技改、富诚铝轮毂、北奔车桥扩能改造、金风风电机组一期、国水投风机塔筒和叶片、二冶风机塔筒、瑞能2兆瓦级风力发电设备和包头北500千伏超高压输变电工程等项目竣工投产，成为拉动工业经济发展新的增长点。优势特色产业全年预计实现工业增加值650亿元左右，对工业经济增长的贡献率达到80%以上。

产业结构不断优化。大力发展新兴产业，神华煤制烯烃项目加快实施，泛海煤化工、东方希望40万吨PVC项目开工建设。华电、龙源、蒙能、鲁能、蛟龙等一批风电项目顺利推进，全市已建成和在建风电项目规模达到99.1万千瓦，其中20万千瓦已并网发电，从风电设备制造到风电场建设的风电产业正在形成。北镁科技、昌欣镁业项目成功试生产，经纬科技、明达镁业、三磊镁合金等镁产业重点项目稳步实施，全市镁合金及深加工企业已达12家。加强重点耗能企业监管，推进企业节能技术改造，坚决淘汰落后产能和工艺，节能工作取得重要进展。加大非煤矿山等资源整合力度，提高了资源综合利用水平。自主创新步伐加快，积极推进企业与高等院校和科研院所产学研联合，大型垂直挤压机设计制造、铝钛硼中间合金、百米高速铁路钢轨、兆瓦级风电机组、非粮生物柴油等重点项目关键技术攻关取得重大突破。全市28家企业被认定为市级以上创新型试点企业。高新技术产业发展规模扩大，我市拥有高新技术企业185户，占自治区的48.2%，预计年内完成工业增加值135亿元，增长30%。

中小企业发展进程加快。实施“一个产业带动百户中小企业工程”和“一个园区带动百户中小企业工程”，新增规模以上中小企业73户，全市中小企业预计实现产值850亿元以上。重点推进中小企业实施装备制造产业配套项目103项，完成投资110亿元以上。积极争取国家、自治区中小企业发展专项资金，集中扶持了17户成长性好的中小企业和3家专门服务中小企业的担保公司。成立6家小额贷款公司，拓宽了中小企业融资渠道。

产业园区实现规模化集约化发展。“园区建设年”活动取得成效，产业园区建设和管理力度加大，承载能力和产业集中度不断提高。稀土高新区发挥全市经济社会发展排头兵作用，实现重大突破，地区生产总值和财政收入增速均超过70%，固定资产投资增长1.47倍。装备制造产业园区实施北奔新型驾驶室、北重安东特殊钢深孔制件等一批装备制造业重点项目，发展态势良好。特钢产业园区入驻企业达到14家，基本形成了从冶炼、热轧、冷轧到制品的较为完整的产业链。包头铝业产业园区入园项目达到38个，化成箔、铝轮毂、铝合金、铝型材及汽车配件实现规模化生产。土右新型工业园区、化工园区和石拐、九原、达茂工业园区也得到长足发展。

（二）着力发展现代服务业，促进第三产业增量提质。预计第三产业增加值完成700亿元以上，增长15%左右。传统服务业日益繁荣活跃。商贸流通项目建设扎实推进，古城湾环保型煤炭装车基地、万号酒店、王府井百货青山店、恒隆酒店等项目投入运营，帝豪天下、东源世纪、中银国际、沃尔玛商业中心等项目顺利实施。全年第三产业固定资产投资突破500亿元，增长40%以上。以神华煤业、呱呱叫、友谊蔬菜等为龙头的50户批发企业，年销售额突破200亿元。以包百、金荣建材、西北汽车城等为龙头的50户零售企业，年销售额突破100亿元。以海德、香格里拉、神华等为龙头的50户住宿餐饮企业，年营业额突破100亿元。小肥羊公司以餐饮业第一名的优异成绩连续三次入围“中国服务业企业500强”，小尾羊公司被评为“中国餐饮连锁业成长十强”企业。我市被商务部认定为自治区唯一的全国加工贸易梯度转移重点承接地。

金融、物流、会展等产业发展实现新突破。引进国内知名银行取得重要进展，浦发银行包头支行正式运营，招商银行、中信银行在包分支机构即将开业。包商银行在外埠设立分行工作取得新成效。全市金融机构本外币存贷款余额分别达到1213.9亿元和632.5亿元，同比增长17.7%和16.4%。“便民服务一卡通”开通，提升了城市信息化水平。钢材、煤炭等生产资料市场迅速发展，全市主要钢材市场交易量达到600万吨，交易金额达到240亿元；煤炭交易量达到8000万吨，交易金额达到220亿元。2008年在我市成功举办了国际多晶硅及光伏产业（包头）峰会、国际铝工业论坛、全国镁行业大会、全国稀土行业重点企业座谈会、自治区首届科技名牌论坛等大型会展活动，包头的知名度和影响力进一步提升。

（三）加快现代农牧业发展，推动“三农”工作取得新成效。预计第一产业增加值完成50亿元左右，增长6%左右。全市用于支农惠农的各类财政专项支出达到6.7亿元，同比增加2.6亿元。转变农牧业发展方式成效明显。加快种植业结构调整，蔬菜保护地新增4100亩。粮食作物获得丰收，总产量预计达105万吨，增产5万吨。设施农业发展加速，全市新增滴灌、喷灌、覆膜种植面积20万亩，累计达到105万亩。达茂旗在全区101个旗县区中率先基本实现全程农业机械化。大力推进畜牧业规模化饲养，建成肉羊养殖村14个、养殖场76个，存栏100头以上的奶牛养殖小区达到148个。

农牧业产业化深入推进。菜薯乳肉等特色产业不断壮大，规模以上产业化加工流通企业实现销售收入185亿元，新增市级以上龙头企业16家。全市有11.5万户农牧民通过参与产业化经营人均收入2016元。品牌农牧业发展势头良好，新增国家级绿色食品7个。种植业保险工作稳步推进，全年投保农作物面积145万亩，同比增加94万亩，投保金额达4100多万元。

新农村新牧区建设扎实开展。85%以上的试点村完成道路、安全饮水等基础设施建设，试点村产业发展和农牧民收入水平进一步提高。全市建成通乡油路和通村公路241公里，新建农村牧区户用沼气池3100座，解决了农村牧区4.1万人安全饮水问题。扶贫开发整村推进工程取得明显效果，全年有16个行政村实施了整村推进项目，解决了0.63万极端贫困人口和1.61万低收入人口的生产生活问题。集中社会力量重点帮扶固阳县工作进展顺利，帮扶资金和物资达到5500万元，落实帮扶项目109项，完成55项，受益农民达到2.66万人。

（四）加强城市建设和环境保护，提升文明城市形象。加强城市规划管理，完成新一轮包头城市总体规划修编工作。全年组织实施了100个城市建设项目，预计完成投资126亿元以上。110国道包头城区段综合改造工程和百灵庙至满都拉口岸、固阳至白云鄂博等一批公路建设项目按计划完工。万水泉大道、建华北路、万青路、青山路、昆北路等道路建成通车。白云鄂博至满都拉铁路开工建设，包头至西安铁路建设和大同至包头、包头至惠农铁路电气化改造工程积极推进。污水资源化利用、城市污水管网改造、青昆垃圾卫生填埋场、热网改造等基础设施工程按期竣工，博奇垃圾发电项目开始实施。会展中心、科技馆、体育中心、图书馆、少年宫建设进展顺利，体育休闲广场、科技少年宫等社会公益项目投入使用。廉租房建设、小街小巷治理、健康水工程等一批便民利民的实事项目圆满完成。石拐棚户区搬迁新建住宅楼主体基本完工，学校、医院等配套设施建设全面推进；包钢尾矿库周边五村搬迁新建住宅楼全部封顶。城市承载和服务功能进一步增强，全市建成区绿化覆盖率达到38%，提高1.6个百分点；集中供热普及率达到81%，提高3个百分点；燃气普及率达到88%，提高3.1个百分点；污水处理率达到81.6%，生活垃圾无害化处理率达到97.2%，人均占有道路面积达到10.9平方米。组建城市管理执法局，加大综合执法力度，整治“六乱”现象，清理违章建筑，城市环境进一步改善。“绿水、青山、大草原”三大生态工程建设取得明显成效，沿黄筑堤修路工程全线开工，大青山南坡绿化已完成工程总量的60%，达茂旗2400万亩草场全部实现围封禁牧，发放围封禁牧补贴9000万元，转移牧民2661户。环境保护工作力度加大，“三片一线”和面源污染整治及企业脱硫设施建设、关停小发电机组等工作取得成效。2008年我市空气质量二级以上良好天数达到295天，增加22天。

（五）推进体制机制创新，继续深化改革开放。从推动城乡统筹协调发展、进一步拓展城区发展空间出发，对全市行政区划进行重大调整。全面推进青山宾馆、包头宾馆等市属企业改革，支持鹿王公司债务重组，为企业减轻债务负担6.6亿元。财政、文化体制改革和人事制度、审批制度改革不断深化。企业上市融资取得新进展，小肥羊公司在香港成功上市。完善农村牧区基本经营制度，土地、草牧场经营水平进一步提高。全面启动集体林权制度改革。建立引导机制，鼓励非公有制经济扩大投资、开发、经营领域，向规模化、品牌化、外向型方向发展，非公有制经济占全市经济总量的比重达到47%以上，市场竞争力显著增强。对外开放进一步扩大，进出口总额完成23.4亿美元，增长27.1%。预计引进国内区外资金达到302.96亿元，增长7.43%；全市利用外资8.15亿美元，增长31.5%。

（六）不断改善民生，全面建设和谐社会。2008年全市用于发展民生事业的资金达到44.6亿元。就业和社会保障工作全面推进。全市城镇新增就业5.49万人，196户“零就业家庭”实现了212名成员就业。农村牧区最低生活保障标准提高600元，达到1300元，低保扩面22%，保障对象增加到5.7万人，补助水平由上年的每人每天1.5元提高到2.74元。城镇居民低保标准提高50元，达到人均每月280元，全年人均月补差达到210元，城乡居民低保标准和补助水平均位居自治区各盟市之首。为全市19.2万离退休人员发放养老金33.2亿元，月人均养老金提高到1201元。全市廉租住房保障资金支出5828万元，11719户低保家庭享受了廉租住房补贴。城区一线清洁工人月工资再次提高200元，达到600元。全面推行城镇居民基本医疗保险，参保率达到87%，参保人数为全区第一。全市分散供养和集中供养的“五保户”供养标准提高到每人每年1200元和1800元，分别增加了400元和600元。调整规范行政机关和事业单位职工、义务教育教职人员以及离退休人员津补贴，提高了企业离休人员和驻包事业单位离退休人员津补贴标准。为保证津补贴及时兑现，市财政补助困难地区专项资金7500万元。

教育、文化和广播电视事业蓬勃发展。基础教育继续保持自治区领先水平。学前教育、职业教育、民族教育、高等教育进一步加强。26所中小学校改扩建工程全部完工；20所中小学校新建工程全部开工，有3所建成投入使用；包头医学院迁入新校区办学。开通了覆盖全市的教育网，教育信息化水平全面提升。对大中专院校贫困生和贫困农牧民子女上学的补贴力度继续加大。广播电视事业全面发展，完成346个20户以上自然村“村村通”工程，广播电视信号综合覆盖率达到98%以上。文化建设全面推进，50%以上的旗县区建成了图书馆、文化馆，迎奥运群众文化活动广泛开展，“鹿城文化艺术节”获得“2008年度中国十佳文化节庆活动”称号。

人民群众健康水平进一步提高。认真解决群众就医难问题，新建、改扩建社区卫生服务站61个、行政村（嘎查）卫生室11个、乡镇（苏木）卫生院19所和县级医院1所。扎实推进新型农村牧区合作医疗制度，新农合参合率提高到95.1%。启动“健康包头行动”，为64200多名困难群众提供医疗资助和义诊服务。全市乡镇（苏木）计生服务站建设基本完成，低生育水平保持稳定，人口和计划生育工作继续走在自治区前列。建成全民健身中心，为200个行政村配备了健身设施。全力支持和备战北京奥运会、残奥会，我市运动员在残奥会上夺得2枚金牌。

社会管理工作继续加强。加大食品药品监督管理力度，妥善处置三鹿婴幼儿奶粉事件，积极排查、医治患病儿童，切实保护奶农利益，努力保持市场稳定，维护了消费者合法权益。救灾抗灾工作得到强化，防凌、防汛、抗旱措施全面落实。全力救援四川地震灾区，向灾区捐赠款物1.4亿元。深入推进立体动态巡控机制，加强社会治安综合治理，保持了社会稳定。加大安全生产监管力度，不断健全完善突发公共事件应急机制。民族宗教、审计、司法、统计、人防、气象、地震、工商、新闻出版、侨务、外事、史志、档案、仲裁等工作进一步加强，为建设和谐包头作出了新贡献。

（七）坚持依法行政，加强民主法制建设。认真执行市人大及其常委会的决议、决定，自觉接受市人大及其常委会的法律监督、工作监督和市政协的民主监督。2008年市政府向市人大常委会报告专项工作8次，向市政协通报工作情况11次。办理人大代表建议126件、政协提案342件，办复率均达100%。加强调查研究，实行重大事项听证制度，提高了政府决策的科学化、民主化水平。全面推进依法治市工作，政府依法行政意识进一步增强。高度重视信访工作，认真开展领导干部大接访活动，集中解决了一批信访突出问题。强化廉政建设，进一步规范行政审批程序，大力推进政务公开，提高了政府工作的透明度。深入开展学习实践科学发展观活动，取得了阶段性成果。

去年四季度以来，面对国际金融危机的影响，我市积极因应新形势、应对新挑战，通过推动工业企业实行电力多边交易、减轻债务负担等一系列政策措施，大力支持各类市场主体克服困难渡难关，同时按照国家新增中央投资的安排方向及重点，努力争取项目和资金，取得了较好成效。

各位代表，过去一年的成绩来之不易。这是在自治区党委、政府和市委的正确领导下，在市人大、市政协的监督和支持下，全市人民团结拼搏、社会各界共同奋斗的结果。在此，我谨代表市政府，向全市人民，向各位人大代表和政协委员，向各民主党派、工商联、无党派人士，向离退休老同志，向中央和自治区驻包单位，向驻包人民解放军、武警官兵、民兵预备役人员和公安干警，向外地来包的投资者、劳动者，向所有关心支持包头改革发展的同志们、朋友们，表示衷心的感谢！

在充分肯定成绩的同时，我们也清醒地看到，我市经济社会发展仍然存在一些突出矛盾和问题。主要表现在：宏观经济下行给我市经济社会发展带来较大影响，部分产业受到严重冲击；调整产业结构和转变发展方式的任务还十分艰巨，节能减排和改善环境质量面临较大压力；产业链中高端项目和规模大的项目不多，高层次创新型人才支撑不足，自主创新能力有待进一步提高；农村牧区基础设施和公共服务体系薄弱，农牧民持续增收的难度加大；中小企业和非公有制经济发展不快；一些地区的项目建设和前期工作相对滞后，等等。对于这些问题，我们将高度重视，在今后的工作中采取切实可行的措施，认真加以解决。

二、2009年主要工作

2009年是新中国成立60周年，也是推进“十一五”规划顺利实施的关键一年。国际金融危机的持续蔓延，对我国实体经济的影响日益明显，我市经济社会发展面临严峻考验。审时度势、应对挑战、增强合力、共克时艰，是政府工作的重大任务。

今年政府工作的总体思路和主要任务是：全面贯彻党的十七大、十七届三中全会和中央、自治区经济工作会议以及市委十届五次全委会议精神，深入落实科学发展观，坚持“科学发展、富民强市”战略，立足扩大内需保持经济平稳较快增长，加快发展方式转变和产业结构优化升级，加快基础设施建设和城乡一体化进程，加快重点领域改革和体制机制创新，加快民生改善和社会事业发展，推进精神文明、民主法制和政府自身建设，努力建设经济繁荣、政治稳定、社会和谐、生态优美、人民幸福的科学发展示范地区。

综合考虑各方面因素，今年全市经济社会发展的主要预期目标是：生产总值增长16%左右，财政收入增长19%左右，固定资产投资增长26%以上，社会消费品零售总额增长18%以上，城镇居民人均可支配收入增长15%左右，农牧民人均纯收入增长15%左右，城镇登记失业率控制在4.5%以内，人口自然增长率控制在5‰以内，完成自治区下达的节能降耗和主要污染物减排指标。

实现上述目标，我们要把握以下原则：一是坚定信心谋发展。既要客观分析、冷静应对我们遇到的困难和挑战，更要看到中央、自治区一系列强有力政策措施带来的发展机遇，看到我市多年来持续健康发展奠定的坚实基础，牢固树立必胜信念，坚定不移地推动经济社会发展。二是强化措施保增长。必须下大力气继续推进项目建设，增加固定资产投资，采取多种方式促进消费，努力扩大出口，保持经济平稳较快增长。三是改善民生见实效。坚持以人为本，把改善民生作为发展的出发点和归宿，千方百计解决好涉及人民群众利益的问题，大力发展社会事业，不断满足人民群众日益增长的物质文化需求。四是维护稳定促和谐。正确处理改革发展稳定的关系，积极应对经济社会发展中出现的新情况新问题，巩固安定团结的大好局面，为推动经济社会又好又快发展营造和谐稳定的环境。

今年要着重做好以下工作：

（一）推进新型工业化，构建富有竞争力的现代工业体系。坚持产业基地化、集群化和高新化发展方向，大力发展现代工业，提升工业经济发展质量。2009年全市拟实施5000万元以上工业重点项目248个，总投资1400亿元以上，年内完成投资480亿元以上；全市规模以上工业增加值增长20%左右。

建设优势产业发展基地。在钢铁产业方面，要抓住国家振兴钢铁、汽车等产业的机遇，推动包钢矿山开发和无缝钢管及高速重轨扩能提质、华业特钢延伸加工等项目的实施，确保钢铁产能及结构调整目标的实现，建设我国中西部地区重要的钢铁基地。在铝产业方面，大力推进包铝和东方希铝相关项目建设进度，实施成基、凯普松、三鑫化成箔二期，国瑞化成箔，桂东电子光箔，吉泰铝型材及稀土铝合金，汇众汽车铝合金零配件二期等一批产业链延伸项目，建设我国重要的铝产业基地。在装备制造业方面，依托一机集团和北重集团的优势，加快实施北奔客车、北方股份工程机械及矿用车、晋西铁路车轴、北创特种作业车等项目，扩大重型汽车、铁路车辆等品牌产品的生产能力，全年重车产能要达到3万辆以上。在电力产业方面，继续加快项目报批和建设，大力发展绿色能源，推动华能、龙源、京能、金杰等一批企业的风电项目尽快实施，力争2009年新建项目规模达到100万千瓦。在煤化工产业方面，加快实施神华煤制烯烃、泛海煤化工、白彦花煤田综合开发项目，积极推进中海油煤化工项目落地。在稀土产业方面，实施包钢稀土高性能磁性材料、新奥核磁共振成像系统、山西通力钕铁硼深加工、广东炜林纳稀土助剂等项目，2009年稀土产业产值要突破100亿元。此外，在硅、镁、塑料等产业方面，要积极推进中冶集团多晶硅、江苏大全多晶硅、山晟新能源光伏材料项目，加快实施四川汉龙氯碱化工和石拐工业园区的一批镁业项目，促进新兴产业做大做强。

推动企业健康快速发展。深入实施“百亿企业培育工程”，力争北重、东方希铝等企业进入超百亿企业行列，使我市超百亿和50亿的企业达到4家和6家。以开展“促进中小企业发展年”活动为契机，大力实施“中小企业升级工程”，使一批中小企业的科技水平得到提升，实力得到增强，成为规模以上企业，逐步形成以大企业大项目为支撑，中小企业协作配套、协调发展的良好格局。加大扶持力度，使本地企业、民营企业优势得到充分发挥。立足于帮助企业应对危机，通过支持企业协作、协调能源价格、保障原材料供应、加强产销衔接、试行政府优先采购制度、企业发展专项贴息、减免或缓缴有关收费等措施，进一步提高企业抗风险能力和可持续发展能力。

加快产业园区建设。加强产业园区基础设施和服务体系建设，推动项目落地，进一步壮大园区实力。稀土高新区要按照科技含量高、产业关联度高、投入强度高、产出密度高的“四高”标准，加快打造以稀土新材料及稀土应用器件为主的高新技术产业集群，建设体制机制创新、节能环保和高效管理的示范区。铝业产业园区、特钢产业园区、装备制造产业园区要加快建设以高附加值延伸项目为主的铝钢深加工产业集群和装备制造企业聚集区。九原工业园区、土右新型工业园区、石拐工业园区、达茂巴润工业园区要进一步强化主导产业，着力引进一批经济效益好、环境污染少的项目，带动产业结构的优化升级。在加快基础设施建设的基础上，积极推进固阳新型特色产业园区建设。

增强自主创新能力。紧紧抓住呼包鄂地区获批成为国家级信息化与工业化融合创新实验区的有利机遇，提升新型工业化发展水平。围绕优势特色产业，重点培育研发一批自主创新产品和技术。加快北重集团超高压厚壁钢管生产的调试步伐，尽快实现产业化；积极推动高强度高韧性贝氏体钢管、高硼铝合金、高性能大吨位旋挖钻机、航天航空用风洞试验装置等项目实施。继续支持企业建立研发机构，今年力争建成铝合金材料工程、钍基核燃料元器件等国家级重点实验室和工程技术中心。加大科技投入，加强创新型人才队伍建设。促进知识产权创造、运用和保护。加强企业与科研院所、高等院校合作，加快科技型中小企业发展，鼓励企业进行科技风险投资，推动高新技术产业化。

（二）继续实施城乡一体化战略，加快农村牧区发展进程。坚持以工业化、城镇化、农牧业产业化推动“三农”工作的发展思路，以工促农、以城带乡，努力形成城乡统筹协调发展的新格局。今年市财政安排农林牧水专项资金2.6亿元，增长34%。

调整优化农牧业结构布局。根据我市各地区农牧业资源禀赋，科学制定发展规划，优化产业布局。近郊地区要围绕市场需求，积极调整种养业内部结构，建设富有鲜明地域特色的农业产业园区，发展优质高效都市农业；远郊地区要推进农牧业规模化、标准化、机械化、专业化，加大农牧业综合开发力度，提高土地产出率、资源利用率和劳动生产率；生态脆弱地区要全面实施“收缩、转移、集中”战略，鼓励农牧民向城镇转移，引导农牧业向生产条件较好的区域集中。

推进农牧业产业化。围绕菜薯乳肉四大主导产业，重点引进和扶持规模大、层次高、带动作用强的龙头企业，加快建设一批专业村、专业户和专业生产基地，提升产业化水平。今年规模以上产业化加工流通企业要力争达到150户以上，销售收入实现200亿元，增长10%以上，参与产业化经营的农牧民达到12万户以上。加快发展多种形式的农牧民专业合作经济组织，完善信息、技术推广等社会化服务体系，提高农牧业的市场化、组织化程度。加强动物防疫、良种繁育和农畜产品质量安全体系建设。扩大农牧业保险覆盖面，增强农牧业抗风险能力。

加强农牧业基础设施和新农村新牧区建设。切实抓好大中型灌区配套续建、节水改造工程，加快固阳县阿塔山水库建设，推动土右旗美岱水库竣工并投入使用。推广滴灌、覆膜、喷灌技术，积极发展设施农业。着力推进农牧业机械化，不断提高农牧业装备水平。扎实开展新农村新牧区建设，鼓励和支持“村企共建”，扶持试点村壮大主导产业。加快完善农村牧区基础设施，今年将建设60公里通乡油路、200公里通村公路和3500座户用沼气池，解决3万农牧民安全饮水问题。

进一步提高农牧民生活水平。推进旗县区工业化进程，壮大县域经济实力。努力稳定农畜产品市场价格，提高农牧业经营效益，增强农牧业产业化对农牧民增收的拉动作用，让农牧民从农牧业中获得更多收益。鼓励农牧民从事第二、第三产业，增加非农产业收入。继续发展劳务经济，引导农牧民依靠外出务工增加工资性收入。发挥建筑业、服务业等劳动密集型产业对劳务输出的带动作用，力争实现劳务输出20万人。认真落实惠农惠牧政策，确保各类补贴及时足额发放到农牧民手中，增加农牧民补贴性收入。积极推进扶贫开发，切实加大投入，增加贫困地区群众收入。继续做好集中社会力量帮扶固阳县工作，今年帮扶资金要达到6000万元以上，搬迁75个自然村，稳定转移农业人口2万人，发展水浇地3万亩，推动帮扶工作取得更大成果。

（三）立足自身特色优势，不断提高现代服务业发展水平。把现代服务业作为推进城市化进程和提升城市竞争力的重要引擎，全面推动第三产业增量提质，努力把我市建设成为我国中西部地区重要的商贸、物流中心和旅游目的地之一。2009年第三产业增加值增长16%以上。

打造现代化区域性商贸中心。完善和提升包百、神华、九星、巴彦塔拉等商务区服务功能。深入推进神华青山一百国际城、金贸东方红商业广场、金基广场、大连万达广场、北梁明清古文化商业步行街等商贸项目实施。在更大范围、更宽领域发展连锁经营、特许加盟、电子商务等现代流通方式，加快超市、专业店、专卖店等新兴业态发展。重点培育小肥羊、小尾羊等地方名优品牌，支持企业开拓国内国际市场。加快发展会计、法律、信息、咨询、广告等现代服务业。继续实施社区商业“双进”工程，积极发展面向广大群众的社区服务业。

大力发展现代物流业。加快生产性物流市场建设，着力打造钢材、有色金属、稀土、化工、机械、煤炭物流基地。积极扶持鹿畅达钢材市场、包头市北方钢材市场和五星物流配送中心等物流企业发展壮大。加快神华物流港、润恒物流园区、新奥蒙华化工物流中心、昕隆不锈钢产品物流中心、满都拉口岸物流园区等项目的实施进度。开工建设神华、盛华铁路专用线、鑫辰煤业美岱煤炭集装站等一批货运场站。完善生活性物流市场功能，促进友谊蔬菜批发市场、佳世隆农贸批发市场、甲尔坝粮食批发市场健康有序发展。推动包头“无水港”开展业务，带动全市及周边地区外向型经济发展。

培育会展、旅游等现代服务业新亮点。办好第三届中国制造业论坛年会和中国稀土产业论坛，积极申办国内外大型会展。加强旅游景区建设，继续完善希拉穆仁草原、九峰山、五当召、美岱召、秦长城、石门风景区等重点景区的基础设施。通过举办节庆活动，加大旅游市场、旅游产品的宣传推介力度，打造精品特色旅游线路，增强我市旅游业的吸引力和竞争力。

（四）抓好节能环保和生态建设，转变经济发展方式。坚持节约资源和保护环境的基本国策，强化生态文明理念，努力建设资源节约型、环境友好型社会，增强可持续发展能力。

加强节能减排和环境保护。采用并推广先进节能技术和工艺，加大重点能耗行业和企业节能管理力度，强化工业节能、建筑节能和社会化节能工作，确保完成节能目标。以创建国家环保模范城市为主线，完善减排“三大体系”，严格控制和削减污染物排放总量。认真执行环境影响评价和环保“三同时”制度，严格控制新建高耗能、低附加值项目，加快淘汰工艺落后和污染严重的产能，为新一轮发展腾出环境容量。引进和实施生物质发电等资源综合利用项目，推动粉煤灰回收利用、冶金废弃物循环利用，加快工业园区污水处理厂建设，提高节能减排水平。进一步抓好城市出入口、南绕城、110和210国道两侧违法排污企业清理整治，巩固“三片一线”治理成果。实施清洁能源替代工程，加快城区燃煤设施整治，优化能源结构，使全市的环境有新的改善。加强水源地保护和水质监测，保障城乡居民饮水安全。2009年全市空气质量二级以上良好天数力争达到300天。

继续加大生态建设力度。巩固围封禁牧成果，全面完成大青山南坡122公里沿线绿化任务。积极推进湿地保护立法工作，完成沿黄筑堤修路57公里的建设任务，启动昭君岛、小白河等六大湿地景点建设。积极推进天然林保护、京津风沙源治理、退耕还林等国家林业重点工程建设，组织实施城郊地区和城乡结合部绿化工程，改善市区外围生态环境。加快非煤矿山资源整合，规范勘查开采秩序，坚决打击私挖滥采破坏环境的行为。大力推进昆河、二道沙河、四道沙河、南海子等河道、水域综合治理工程。结合黄河减灾泄洪工程，做好引水入城项目的前期工作，为全市可持续发展提供有力支撑。

（五）坚持改革开放，进一步增强发展活力。继续解放思想，推进改革创新，着力构建充满活力、富有效率、更加开放、有利于科学发展的体制机制。

推进重点领域改革创新。深化农村牧区各项改革。按照依法自愿有偿的原则，推进农村土地承包经营权有序流转，引导发展适度规模经营。全面推进集体林权制度改革，基本完成商品林及宜林地的确权工作。优化国有经济布局和结构，推动国有资本向优势产业及关键领域集中。鼓励、支持和引导非公有制经济发展，为企业创造公平竞争的政策环境、市场环境和法制环境。认真做好小尾羊公司等重点企业上市工作，扩充拟上市企业后备资源。深化公用事业改革，进一步完善发挥市场作用与保障群众利益相结合的发展体系。加快文化体制改革，增强文化事业发展活力。积极推进事业单位分配制度改革。进一步完善人才培养引进政策和工作机制，全面推进人才集聚市建设。

扩大对外开放。进一步优化投资结构，吸引外商投资向高新技术产业、高附加值产品制造、高端研发及设计领域拓展。全年实际利用外资力争达到9亿美元。抓住发达地区特别是沿海地区制造业向内陆地区转移的机遇，以珠三角、长三角、京津冀等地区为重点，加大招商引资力度，努力扩大引资规模。2009年引进国内区外资金要达到330亿元左右，增长10%。优化进出口商品结构，积极引导加工贸易转型升级。鼓励企业“走出去”开展工程承包、劳务、技术服务等国际合作。继续实施向北开放战略，积极开展对俄蒙经贸合作。大力发展口岸经济，加强与蒙方沟通协调，推动口岸基础设施建设，把满都拉口岸建设成为我国对俄蒙贸易的重要通道。

（六）加强城市建设管理，全面提升承载能力和服务功能。围绕建设国家生态园林城市目标，按照“新区开发，旧城提升，城乡统筹，整体推进”的思路，坚持高起点规划、高水平建设、高效能管理，努力建设布局合理、设施配套、功能齐全、环境优美的现代宜居城市。

继续完善城市基础设施。2009年实施城建项目169个，总投资415亿元，年内完成投资240亿元。加快建设站前路、沼潭东路、机场高速西延立交桥等道路、桥梁工程项目，实施集中供热管网扩建、滨河新区热源厂、城乡配电线路、天然气加气站和中高压管网建设等一批供热供电供气工程，积极推进市区和旗县区污水处理厂建设，切实增强城市服务功能。继续推进健康水工程，新增受益人口20万。实施市容综合整治工程，完成33条小街小巷综合治理和20条道路“五化”建设；改造10个旧住宅小区和43处“夹心房”；新装路灯7000盏；新建公厕20座，全市所有公厕免费开放；新建和改造市区垃圾转运站60座。实施公交便民工程，更新购置大容量、环保型公交车辆50台；新建、改造停靠站20处，候车廊及站牌100座；在部分干道开通公交专线，调整、延伸部分公交线路。启动包头市植物馆和城市规划展览馆项目，推动保利园林式国宾馆和体育休闲公园投入使用。加大旧城改造力度，加快北梁地区开发。全面完成石拐棚户区、包钢尾矿库周边五村搬迁移民新区及配套工程建设，努力让搬迁居民早日入住新居。实施城乡结合部改造工程和10个“城中村”改造项目，推进阿东热源厂、市殡仪馆及公墓搬迁工作，改善城市环境。加强小城镇建设，进一步发挥小城镇集聚产业、人口及各类生产要素的重要作用。加快滨河新区、新都市区和昆区南部区开发，使之成为我市提升城市品位、完善服务功能、增强中心城区辐射带动力的新亮点。

加快大交通网络建设。协同有关方面实施西南绕城高速公路、包头至鄂尔多斯树林召高速公路和京藏高速公路呼包段改造项目，优化我市高速公路路网结构。加快110国道包头西北门至哈业胡同、东兴至萨拉齐段公路和省道211线固阳至百灵庙公路改造，建设省道104线百灵庙至白云鄂博、市区北出口至固阳明登、市区至石拐一级公路；同时实施一批农村牧区公路建设项目和公路运输枢纽建设项目。通过以上项目的实施，市区与外五旗县区实现一级公路联通。加快集宁至包头第二双线铁路和包头至西安铁路建设，积极推进包满铁路项目实施。启动机场候机楼、停机坪改造工程，争取年内包头机场进入百万级干线机场行列。做好新机场规划选址等前期工作，为下一步机场迁建奠定基础。开展城市轨道交通项目调研论证和相关前期工作，适时推进项目建设。

继续加强城市管理工作。进一步提高城市规划区内控制性详规的覆盖率，做好重点地区、重点地段的城市设计和建筑精品设计，精心打造城市亮点。强化市政、园林设施管护工作，确保公用设施的正常高效运转。有效整合城市管理资源，进一步完善城管综合执法体制机制，促进城市管理科学化、制度化、规范化。加大对城乡结合部和拆迁改造区域的整治力度，坚决制止和打击私搭乱建等违法行为。加强村镇规划管理，促进城市管理向农村牧区延伸。做好社区管理和服务工作，把社区建设成为管理有序、服务完善、文明祥和的社会生活共同体。

（七）提高财税金融的保障能力，强化措施扩内需保增长。把握国家实施积极财政政策和适度宽松货币政策的机遇，集中财力落实扩内需、保增长、重民生的各项措施，促进经济平稳较快发展。

扎实做好财税工作。抓好财源建设，认真做好增值税转型相关工作，减轻企业负担，增强企业抗风险能力和可持续发展能力。大力培植新的税收增长点，完善财税收入增长监测分析机制，加强税收征管，堵塞漏洞。优化收支结构，努力提高财政收入占地区生产总值和税收占财政收入的比重，加大对“三农”、教育、社保和就业、医疗卫生、环境保护等公共服务领域的投入。坚持勤俭节约，集中财力办大事。为积极应对国际金融危机，政府将为企业协调银行贷款并给予贴息支持，今年市区两级财政对中小企业贷款贴息要达到1亿元。

增强金融对经济增长的助推作用。金融是现代经济的核心。各金融机构都要按照国家政策的要求，积极与企业对接，保证信贷资金畅通地流向企业、用到实体经济。提高金融机构对中小企业的贷款比重，鼓励小额贷款等民间资本进入，切实缓解中小企业贷款难、融资难问题。制定全市金融业发展规划，完善政策措施，鼓励和支持商业银行增加信贷投放，扩大信贷规模。推动渤海、民生、光大、兴业等股份制银行在我市设立分支机构，进一步繁荣我市金融市场。支持包商银行发展壮大。加快组建农村商业银行，推进村镇银行发展，为农村牧区发展提供资金保障。加强社会信用体系建设，改善信贷环境和金融业发展环境。

扩大投资和消费需求。加大固定资产投资力度，充分发挥项目建设对经济增长的拉动作用。大力引进和实施符合国家产业政策、有利于提升产业层次的非资源型项目。认真做好重点项目规划、论证、申报、立项等前期工作，加强与国家、自治区有关部门的对接，争取项目审批和资金支持。同时要积极落实企业投资，鼓励社会投资，加快项目建设。2009年，我市拟实施投资5000万元以上重点项目592个，项目总投资达2738亿元，年内计划完成投资941亿元。制定和实施拉动消费的政策措施，促进消费结构升级，进一步扩大消费规模。引导和鼓励居民扩大住房消费，对我市居民购买120平方米以下普通住房给予一定的财政补贴。大力培育居民在通信、文化、旅游、体育等方面的消费新热点。加快“万村千乡”市场工程建设，积极实施“家电下乡”工程，不断完善农村牧区市场体系，扩大农村牧区消费。

（八）高度关注民生，切实维护好人民群众的根本利益。坚持把实现好、维护好、发展好最广大人民群众的根本利益作为政府工作的出发点和落脚点，努力提高人民群众的生活水平和生活质量，切实做到发展为了人民、发展依靠人民、发展的成果由人民共享。今年，市本级财政安排资金9.8亿元用于保障和改善民生，同比增加1.5亿元，占全部新增财力的38.4%。

千方百计扩大就业。研究制定促进就业的相关政策，建立支持自主创业、自谋职业的激励机制，以创业带动就业。完善公共就业服务体系，加强技能培训和有针对性的就业指导服务。今年争取城镇新增就业5.5万人。积极引导企业履行社会责任，鼓励企业吸纳本市劳动力就业。支持遇到困难的企业采取在岗培训、轮班工作等办法，稳定现有就业岗位，尽量不裁员、不降薪。建立企业裁员备案、农民工返乡统计报告等制度，加强失业调控和预警。加大对“零就业”家庭、4050人员及其他就业困难群体的就业援助和生活救助力度。鼓励大学生自主创业或到农村牧区工作。鼓励农牧民进城创业，对返乡创业的农民工给予政策扶持。启动实施对农村牧区“零转移”家庭的专项援助行动。

建立健全覆盖城乡居民的社会保障体系。全面推行城乡居民基本养老保险制度，做好私营企业员工、个体劳动者等未参保人员养老保险补办工作，实现养老保险政策的全覆盖。继续提高企业退休人员养老金标准并按时足额发放。切实解决部分企业职工以及灵活就业人员未能参保的问题，更好地实现医疗保险对城镇职工的全覆盖。进一步提高新型农村牧区合作医疗补助标准，达到每人每年120元。为全市40岁以上的农牧民免费体检。城镇居民低保标准提高30元，达到人均每月310元；农村牧区居民低保补助水平每人每年提高180元，达到1180元。提高城镇低保家庭取暖补贴200元，达到每户每年600元。继续提高环卫一线清洁人员工资，达到每人每月800元，并按有关规定缴纳养老、医疗和工伤保险。提高全市中小学班主任津贴标准，提高市级劳动模范和先进生产（工作）者待遇。加大对困难家庭大中专学生资助力度。做好农村牧区“五保户”供养、灾民救济工作。抓好失业保险和生育保险工作。加大对残疾人扶助力度，实施“百户安居”、“千人参加职业技能培训”、“万名残疾人享有康复救助服务”工程。加强保障性住房建设，年内新开工建设120万平方米经济适用房，建设15万平方米廉租住房，使人均住房建筑面积不足13平方米的低保家庭都能享受廉租住房。

切实维护人民群众合法权益和社会安全稳定。整顿和规范市场秩序，依法惩处制假售假、商业欺诈等行为，保障消费者合法权益。深入开展食品药品安全专项整治，加强对生产流通过程的监管，防止不合格产品进入市场。加强房地产市场和建筑市场监管，促进房地产市场持续稳定健康发展。狠抓非煤矿山、尾矿库、沉陷区等重点领域的安全监管，关闭不符合安全生产条件的企业，坚决遏制重特大事故发生。加强城市防灾体系建设，建立健全社会预警体系和应急救援、动员机制，提高处置突发事件能力。加强社会治安综合治理，进一步完善社会治安防控体系，依法打击各类违法犯罪活动。高度重视信访工作，继续深入开展领导干部大接访活动，妥善解决信访突出问题，切实维护人民群众利益。

2009年，市政府将围绕社会保障、公共服务、改善环境等方面，继续为人民群众兴办20件48项好事实事，总投资60多亿元。这是市政府对全市人民的郑重承诺。我们将以这些好事实事为重要抓手，把更多的人力、物力、财力投入到保障和改善民生上来，让人民群众在改革发展中得到更多实惠。

（九）加强社会建设，促进各项社会事业全面发展。巩固文明城市建设成果，大力推进公共服务均等化，建设教育强市、文化大市和体育大市，努力提升全体市民的综合素质，实现经济、社会与人的全面协调可持续发展。

办好人民满意的教育。优化配置基础教育资源，大力推进素质教育。全面完成20所学校续建任务，改扩建20所农村牧区中小学校，实施50所中小学校标准化建设，进一步改善全市中小学办学条件。优化教育教学内容和方法，减轻中小学生课业负担。加强师德师风教育，提高教师队伍素质。加大教育投入，逐步推进高中阶段免费教育，义务教育和普通高中教育要继续保持自治区领先水平。实施民族教育发展工程，改扩建12所民族中小学，全面提高办学质量。建设包头市特殊教育学校康复中心，为残疾儿童和智障儿童提供更好的教育。整合全市职业教育资源，对我市管理的15所中职学校进行重点整合，重组建设5所具有特色的职业技术学校，扩大原有6所高职院校办学规模。创造条件做好包头稀土学院的报批组建工作。重视高等教育，发展全民终身教育。

加快文化大市建设步伐。加强基层文化设施建设，今明两年全部完成旗县区文化馆、图书馆和34个乡镇苏木综合文化站新建改扩建工程。全面推进社区和行政村（嘎查）基层文化室建设。完善文化大市建设规划，开工建设包头文化艺术中心，鼓励社会投资建设专题博物馆。办好鹿城文化艺术节，创建一批各具特色的文化活动品牌。注重引进和培养文化专业人才，扶持文化精品创作，编排一批优秀剧目，为建国60周年献礼。今年全市艺术团体送戏下基层演出1000场以上，农村牧区电影放映达到8000场。保护和弘扬民族文化遗产，加快推进王若飞纪念馆治理改造工程，继续做好美岱召、五当召、转龙藏龙泉寺等文物古迹的修缮保护工作，启动燕家梁遗址博物馆项目，建立我市非物质文化遗产保护名录。推动广电事业发展，开工建设包头市广播电视中心和广播电视多功能发射塔。加快公共文化服务体系建设，大力推进文化事业产业化。加强广场文化、社区文化、乡村文化、校园文化和企业文化建设，繁荣本土文化和民族文化，丰富市民精神文化生活。

提高人民群众健康水平。加强基层卫生设施建设，完成10个社区卫生服务中心和60个行政村（嘎查）卫生室的新建和改扩建工程，为45个已建成的乡镇卫生院配置医疗设备。新建包头市妇幼保健院、中心血站、卫生监督所综合业务大楼，改善旗县区妇幼保健所的基础条件。建成七医院外科系统综合楼、包钢医院综合住院楼、北重集团医院综合病房楼，改善医疗服务环境。强化农村牧区和城市社区卫生人才队伍建设。规范医药市场管理。做好重大传染病防控工作。继续深入开展“健康包头行动”。落实好人口和计划生育政策，提升计生服务水平。加强体育基础设施建设，新建市体校和乒羽球馆、网球中心等一批体育专业场馆，完成2010年世界中学生排球锦标赛主要场馆建设，为全市266个行政村配备体育健身设施。提高竞技体育水平，积极备战自治区第十二届运动会，办好我市第十一届运动会和少数民族运动会。

推动精神文明建设和社会全面进步。深入开展群众性精神文明创建活动，提高全社会文明程度。积极支持国防建设，加强军政军民团结，继续深入推进双拥模范城建设。做好民族宗教工作，发展平等团结互助和谐的社会主义民族关系，争创全国民族团结进步模范市。加强普法工作，提高依法治市水平。完成第二次经济普查主要任务和第六次人口普查前期准备工作。认真做好老龄工作，支持工会、共青团、妇联等群团组织开展工作。全面发展审计、外事、侨务、地震、气象、档案、史志等各项事业。

（十）加强政府自身建设，提高公共管理服务能力和水平。按照社会主义政治文明建设要求，以建设公共服务型政府为方向，推进政府管理创新。

转变政府职能。全面履行经济调节、市场监管、社会管理和公共服务职能，为市场主体创造良好发展环境。进一步深化行政审批制度改革，积极推进“网上审批”和“一站式服务”，优化政务环境。加强服务型政府建设，完善社会管理和公共服务体系，为群众、企业、基层提供优质便捷服务。

坚持依法行政。严格按照法定权限和程序行使权力、履行职责、接受监督，保证人民赋予的权力始终用来为人民谋利益。自觉向人大及其常委会报告重大事项，认真执行人大及其常委会的决议和决定，积极支持政协履行政治协商、民主监督和参政议政职能。重视社情民意信息，认真办理人大代表建议、批评、意见和政协提案，广泛听取各民主党派、工商联、无党派人士的意见建议，主动接受新闻舆论和社会公众监督。

改进工作作风。坚持群众路线，深入开展调查研究，扩大政务公开范围，切实保障群众的知情权、参与权、表达权和监督权。加大督查力度，提高办事效率。紧紧抓住国家实施扩大内需一系列措施的重大机遇，结合我市实际，为企业发展服务，为项目建设服务，不断增强推动经济平稳较快增长的合力。

加强廉政建设。坚持标本兼治、综合治理、惩防并举、注重预防的方针，认真落实党风廉政建设责任制，严格执行领导干部廉洁自律的各项规定。深入开展治理商业贿赂工作，坚决纠正部门和行业不正之风，着力解决群众反映强烈的突出问题，努力让人民群众满意。

各位代表，在新的起点上实现包头的新发展，是时代和全市人民赋予我们的光荣使命。展望未来，我们充满信心。让我们紧密团结在以胡锦涛同志为总书记的党中央周围，在中共包头市委的领导下，高举中国特色社会主义伟大旗帜，坚持以邓小平理论和“三个代表”重要思想为指导，深入贯彻落实科学发展观，解放思想，开拓进取，为建设富裕民主文明和谐新包头努力奋斗！
